# *In planta* complementation of the betalain biosynthetic pathway with a bacterial dioxygenase

**DOI:** 10.1371/journal.pone.0325603

**Published:** 2025-06-24

**Authors:** María Alejandra Guerrero-Rubio, Hester Sheehan, Samuel F. Brockington

**Affiliations:** Department of Plant Sciences, University of Cambridge, Cambridge, United Kingdom; University of Illinois at Urbana-Champaign, UNITED STATES OF AMERICA

## Abstract

Betalains are pigments naturally produced by plants belonging to the order Caryophyllales. However, 4,5-L-DOPA-extradiol-dioxygenase (DODA), the main enzyme in their biosynthetic pathway, has also been characterized in other kingdoms including fungi or bacteria. These enzymes show few similarities with the plant enzymes at structural or kinetic levels but achieve the same activity, raising questions on their possible exchange with plant DODA enzymes and subsequent complementation in the biosynthetic pathway of betalains. In this work, we show that the DODA enzyme from the bacterium *Gluconacetobacter diazotrophicus* is able to provide the activity of L-DOPA dioxygenase and produce betalamic acid and betalains, *in planta*. Betalains were detected after transient expression of the genes required for their synthesis in the plant model *Nicotiana benthamiana*. HPLC-ESI-MS analysis showed that the expression of the bacterial enzyme gave rise to multiple betalains in a similar manner to plant DODAs, with vulgaxanthin I being the main pigment obtained in infiltrated leaves. Our results show that fully functional betalamic-forming enzymes from outside of the plant kingdom are able to complement the plant-based betalain biosynthetic pathway. This supports the proposition of convergent evolution of enzymes with betalain-forming activity, not only within Caryophyllales, but also in other kingdoms.

## Introduction

Betalains are pigments that provide coloration to plants in the order Caryophyllales, excluding six families [1–3] where anthocyanin pigmentation is retained. Betalains and anthocyanins are mutually exclusive pigment classes and have never been found together in the same plant [4]. Betalains have attracted increasing interest as natural pigments, with antioxidant, antitumoral and anti-inflammatory properties [5]. Their health-promoting effects have led to the study of their biological capacities *in vitro* and *in vivo* and the biosynthetic pathway has been expressed in heterologous hosts such as cotton [6], carrots [7], eggplants and tomatoes [8]. Among the enzymes responsible for betalain pigmentation, is the core committed enzyme, 4,5-L-DOPA-extradiol-dioxygenase (DODA).

**Fig 1:**
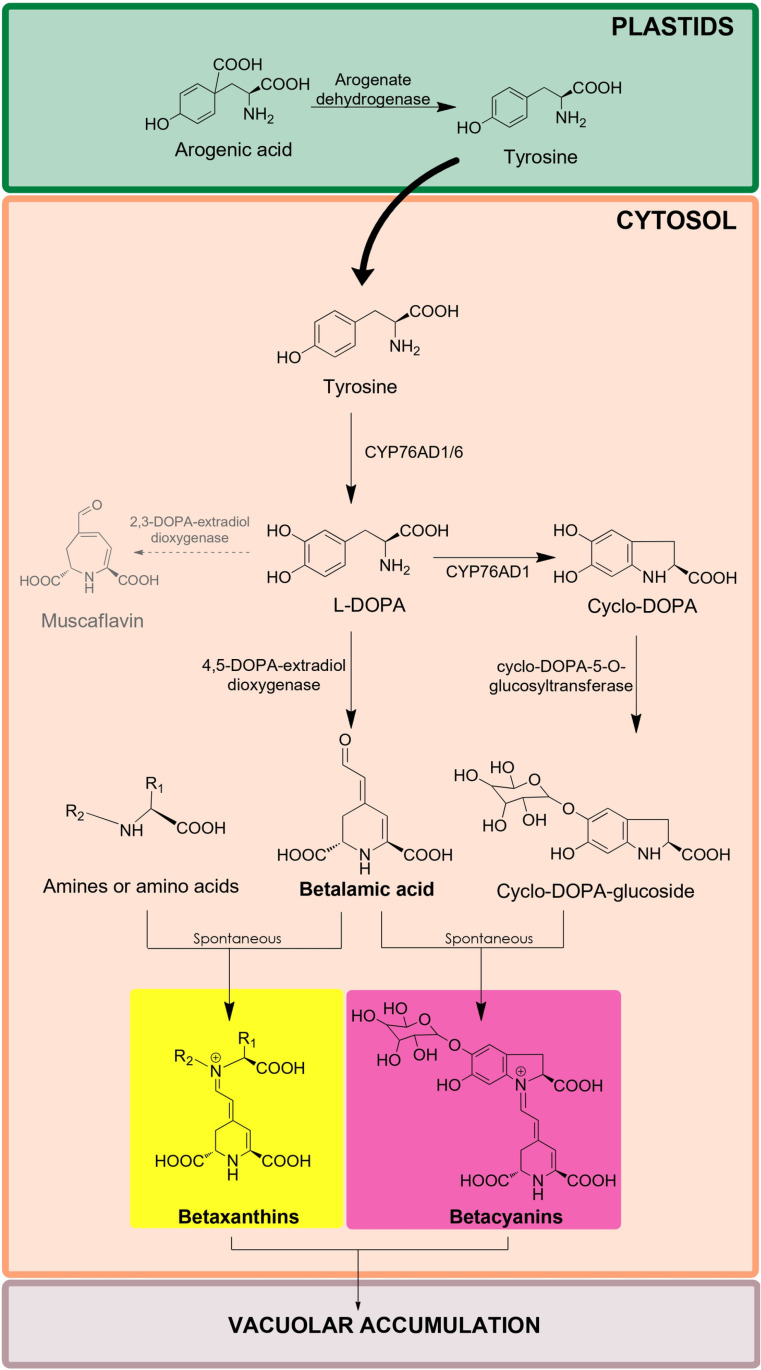
Schematic representation of the metabolic pathway of betalains in plant cells. Enzymatic and spontaneous reactions are performed in the cytosol to produce yellow betaxanthins and violet betacyanins that are transported to the vacuole for storage. In grey, represents the production of muscaflavin due to the additional 2,3-L-DOPA-extradiol-dioxygenase activity performed by *G. diazotrophicus* DODA.

The DODA enzyme is responsible for the cleavage of L-DOPA to yield betalamic acid, the central chromophore and structural unit of all betalains. In plant cells, betalamic acid is produced in the cytosol where it condenses spontaneously [9,10] with amino acid or amines to form yellow betaxanthins, or with indole-derived molecules such as cyclo-DOPA to produce violet betacyanins. Both betaxanthins and betacyanins are subsequently transported to vacuoles for storage [11,12] (Fig 1). The DODA enzymes in plants are an example of convergent evolution as betalain-producing organismal lineages do not all share a most recent common ancestor. In fact, there is evidence to state that DODA arose independently at least four times within betalain-producing species [13]. The presence of these enzymes in Caryophyllales plants seems to be derived from several, independent events of duplication and neofunctionalization. Interestingly, deeper levels of convergence are apparent with enzymes with betalamic acid-forming activity that have also been described in fungi *Amanita* [14] and *Hygrocybe* [15], in the cyanobacterium *Anabaena cylindrica* [16] and in the bacterium *Gluconacetobacter diazotrophicus* [17].

Among these enzymes outside the plant niche, GdDODA has attracted attention for the production of betalains. This singular enzyme present in the sugarcane symbiotic bacterium *G. diazotrophicus* presents dual activity 2,3- and 4,5-L-DOPA dioxygenase. Thus, GdDODA not only produces betalamic acid from L-DOPA but also muscaflavin due to the 2,3-ring cleavage activity (Fig 1). GdDODA has been employed to obtain betalains in microbial biofactories [18] due to its remarkable *in vitro* activity. Comparing its *in vitro* kinetic parameters (Km = 1.36 ± 0.31 mM, Vmax = 5.26 ± 0.43 µM · min-1) to those from the BvDODA enzyme isolated from the betalain-producing plant *Beta vulgaris* [19] (Km = 6.9 ± 0.9 mM, Vmax = 1.2 ± 0.1 µM · min-1), demonstrates that GdDODA is more efficient in producing the ring cleavage of L-DOPA to produce betalamic acid [19]. Additionally, these first studies with BvDODA enzyme showed that the enzyme was not efficient in the production of betalains in microbial hosts. More recent phylogenetic analysis performed with Caryophyllales plants have classified the aforementioned enzyme from *B. vulgaris* as DODAα2, an isoform that does not produce betalains efficiently but presents marginal activity. Rather, the paralog DODAα1 is the isoform directly involved in the betalain pigmentation since DODAα1 exhibits high affinity for L-DOPA to be used as substrate in the production of betalamic acid [13].

In the present study, we compare the activity of the bacterial enzyme from *G. diazotrophicus* with the activity of a plant DODAα1 enzyme to produce betalains in *Nicotiana benthamiana* plants, a plant species widely used as model for many plant molecular studies [20]. *N. benthamiana* has previously been used in studies related to the heterologous expression of the main enzymes involved in the betalain pathway (Fig 1) such as arogenate dehydrogenase (ADH), the precursor of tyrosine synthesis [21], CYP76AD6, which yields L-DOPA from tyrosine, and CYP76AD1, which catalyses both L-DOPA formation from tyrosine and its conversion to cyclo-DOPA- [22], DODA, whose main activity has already been described, and cDOPA-5GT, which uses cyclo-DOPA as substrate to produce cyclo-DOPA-glucoside [21]. The results obtained in this field have also allowed the use of plant betalain biosynthetic genes as simple and effective reporters as the violet pigment betanin is easily detectable after infiltration of multigene-assembled constructs in leaves [23] or roots [24]. However, the relative performance of bacteria-derived and plant-derived DODAs have never been evaluated *in planta*. Here, we show for the first time how a bacterial enzyme is capable of substituting for the activity of a plant dioxygenase enzyme to complement the betalain biosynthetic pathway, albeit with more limited efficacy.

## Materials and methods

### Strains and growth conditions

*Escherichia coli* DH5α was employed for its transformation with the different produced plasmids. *E. coli* culture was grown at 37°C in Luria Bertani (LB) media supplemented with spectinomycin 50 mg/mL, ampicillin 50 mg/mL or kanamycin 50 mg/mL for the selection of plasmids in the Golden Gate cloning assay (MoClo) [25] at the so-called Level 0, Level 1 or Level 2, respectively. *Agrobacterium tumefaciens* GV3101 was grown in LB media at 30°C and supplemented with kanamycin (50 mg/mL), gentamicin (25 mg/mL) and rifampicin (50 mg/mL). *A. tumefaciens* harbouring multigene vectors was employed for its infiltration in *N. benthamiana* plants. *Nicotiana benthamiana* plants were grown in soil under long day conditions (16 h light; 8 h dark) in controlled growth rooms maintained at 20°C with 60% humidity.

### *Gluconacetobacter diazotrophicus* DODA sequence and cloning

The gene sequence of *G. diazotrophicus* (sequence WP_012222467.1) was used as a template to synthetically obtain the DODA sequence from *G. diazotrophicus*, optimized for *N. benthamiana* expression. PCR amplification was performed using Phusion High-Fidelity DNA polymerase (Thermo Fisher Scientific, Waltham, MA, USA) and the following primers: GdDODAco_Lv0_F (5′TGAAGACATAATGACACCAGTTCCCGAAC3’) and GdDODAco_Lv0_R (5′TGAAGACATAAGCTCATATAGGTGTAGCTCCCCC3’). This amplification yielded a PCR product of 429 bp which was extracted from agarose gel with PureLink Quick Gel Extraction Kit (Invitrogen, Carlsbad, California, USA) and subsequently used for further experiments.

### Production of vectors for betalain synthesis

**Fig 2:**
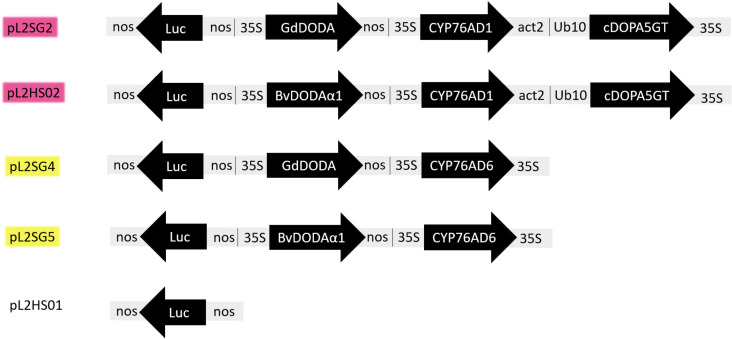
Multigene vectors employed in this work. Multigene vectors were built by using the modular cloning technique to reconstruct the betalain biosynthesis pathway. Yellow pigments, betaxanthins, are obtained by the activity of CYP76AD6 and DODA enzymes. Violet pigments, betacyanins, are obtained by the expression of CYP76AD1, cDOPA5GT and DODA. Name of obtained plasmids are highlighted to indicate if they were designed to produce betacyanins (violet), betaxanthins (yellow) or as a control (no background).

The construction of multigene vectors containing the betalain biosynthetic genes was carried out using Golden Gate cloning. Cloning components were acquired from the MoClo Plant Tool Kit and the MoClo Plant Parts Kit (Addgene, Cambridge, MA, USA) and coding sequences of genes (S1 Table) involved in the betalain pathway were codon optimised for their expression in *N. benthamiana*. Cloning proceeded through Level 0, Level 1 and Level 2 modules using the long one-pot, one-step Type IIS mediated cloning reaction previously described [26]. Constitutively expressed promoters and terminators were used for all Level 1 transcriptional units: *PpLUC* under control of the *nos* promoter and terminator; *GdDODA* under control of the long *CaMV 35S* promoter and the *nos* terminator; CYP76AD1 and CYP76AD6 under control of the long *CaMV 35S* promoter and the *A. thaliana* actin 2 (*act2)* terminator; *cDOPA5GT* under control of the *Ub10* promoter and *35S* terminator. All level 2 vectors employed in this work are shown in Fig 2. The level 2 vector pL2SG2 was designed with transcriptional Level 1 units in the following positional order: *PpLUC* (reverse orientation); *GdDODA; CYP76AD1; cDOPA5GT*. The level 2 vector pL2SG4 was designed in the following order: *PpLUC* (reverse orientation); *GdDODA;* and *CYP76AD6* units. The level 2 vector pL2SG5 was designed in the following order: *PpLUC* (reverse orientation); *BvDODA;* and *CYP76AD6.* Level 0 and Level 1 employed for the design of these multigene vectors were confirmed by sequencing of the full inserts. Level 2 vectors were confirmed by restriction enzyme digests following a confirmation by sequencing the boundaries of the insert. Sequencing services were provided by Source BioScience (Nottingham, UK) and the obtained sequences were analysed using Geneious software (Biomatters, Auckland, NZ).

### Transient expression of GdDODA in *Nicotiana benthamiana*

Transient gene expression assays in *N. benthamiana* were performed according to the previously described agroinfiltration method with some modifications [27]. All constructs were transformed into the *Agrobacterium tumefaciens* GV3101 strain and grown in LB media supplemented with kanamycin (50 mg/mL), gentamycin (25 mg/mL) and rifampicin (50 mg/mL). Cultures were grown at 28°C with orbital shaking until reaching an OD_600_ = 1.5. Cultures were centrifuged 10 min at 10,000 rpm, supernatants were discarded and pellets were diluted to a final OD_600_ = 0.5 in infiltration media (10 mM MgCl_2_, 0.1 mM acetosyringone, 10 mM MES at pH 5.6). Cultures were maintained at room temperature for 2–3 h before infiltration. Infiltration was performed on 6-week-old *N. benthamiana* plants and six individual plants were employed as independent biological replicates. Infiltration in young leaves was facilitated by first generating a small shallow cut on the adaxial leaf surface. Infiltrated tissue was sampled 4 days post-infiltration from the five biological replicates for betalains identification and quantification.

### Betalain extraction

For each infiltration, 25–40 mg of fresh weight leaf tissue was sampled and immediately frozen in liquid nitrogen in 15 mL tubes. Sampled leaf tissue was ground frozen using a Tissue Lyser II homogeniser (QIAgen, Hilden, Germany). Betalains were extracted overnight at 4°C in 20 mM phosphate buffer pH 6.0, 50 mM sodium ascorbate with a volume of 1 mL extraction buffer per 50 mg leaf tissue. After extraction, the samples were centrifuged twice for 10 min at 10,000 rpm and the final supernatants were collected for further analysis.

### Spectrophotometric analysis of *N. benthamiana* leaves

Extracts from infiltrated leaves were employed for betalain quantification. 100 μL of each supernatant was transferred to a black μCLEAR 96-well microplate (Greiner Bio-One, Kremsmünster, Austria). Absorbance and fluorescence of betalains were measured for each well with a CLARIOstar microplate reader (BMG Labtech, Aylesbury, UK). Absorbance values were measured between 400–700 nm with a resolution of 5 nm. Betacyanin relative concentration was calculated as *A*540 − (0.12 × *A*660) where A540 and A660 are the absorbance values for betacyanins and chlorophyll *a* at 540 nm and 660 nm, respectively. Fluorescence was measured using an excitation wavelength of λ = 470 nm and emission wavelength of λ = 510 nm.

### Betalain quantification

Pigment-containing extractions from *N. benthamiana* leaves were then employed to quantify the total content in betaxanthins or betacyanins by following an end-point method that measures the degradation of the betalain, yielding free betalamic acid detected at 424 nm. A Jasco V-650 spectrophotometer (Easton, MD, USA) was used to perform absorbance measurements in the 400−700 nm range. Samples were subjected to hydrolysis using ammonia diluted 1:50 in water. The degradation processes were monitored for 30 min, performing spectra every 2 minutes and the resulting betalamic acid solutions provided the concentration of each sample. The molar absorption coefficient (Ɛ) of 48,000 M^-1^ cm^-1^ was employed for betaxanthin quantification and Ɛ = 65,000 M^-1^ cm^-1^ was employed for betacyanin quantification.

### Fluorescence microscopy

Images from leaves expressing fluorescent betalains were taken at constant exposure times using filtercube I3 and a 20x lens in a Leica DM 2500 LED microscope fitted with a Leica DFC550 camera (Leica Microsystems, Wetzlar, Germany) with an incident light beam.

### HPLC analysis

A Shimadzu LC-10A apparatus (Kyoto, Japan) equipped with a SPD-M10A PDA detector was used for analytical HPLC separations. Reversed phase chromatography was performed with a 250 x 4.6 mm Kinetex 5μm C-18 column (Phenomenex, Torrance, CA, USA). Gradients were formed with the following solvents: solvent A was water with 0.05% trifluoroacetic acid (TFA), and solvent B was composed of acetonitrile with 0.05% TFA. A linear gradient was performed for 25 min from 0% B to 35% B. The flow rate was 1 mL/min, operated at 25°C. Injection volume was 50 μL. Pure betalains were included as controls using their retention times and maxima wavelengths in order to identify known betalain compounds. A volume of 50 μL of each sample was employed for HPLC analyses. Chromatogram at λ = 405 nm was used to determine the presence of betalamic acid and muscaflavin, λ = 480 nm was used for betaxanthins detection and λ = 536 nm was used to follow betacyanins.

### Betalain standards

Pure betalains were employed as standards in HPLC analysis in order to identify the different peaks obtained in post-infiltrated *Nicotiana* samples. Betanin was extracted from *B. vulgaris* roots [28], betanidin was extracted from *Lampranthus productus* and the remaining betalains employed were obtaining by biotransformation in *E. coli* cultures containing the 4,5-DOPA-extradiol-dioxygenase enzyme from *Gluconacetobacter diazotrophicus* (GdDODA), following the method described by Guerrero-Rubio et al. 2019 [18]. Purification of samples was performed with a combination of different chromatographic steps as described by Gandía-Herrero et al. 2006 [29].

### Electrospray ionization mass analysis

An Agilent VL 1100 apparatus with LC/MSD Trap (Agilent Technologies, Palo Alto, CA, USA) was used for HPLC–ESI–MS analyses. Elution conditions were the same as described in the HPLC analysis section, using a Kinetex 5μl C-18 column with a flow rate of 0.8 mL/min. Vaporizer temperature was 350°C, and voltage was maintained at 3.5 kV. The sheath gas was nitrogen, operated at a pressure of 45 psi. Samples were ionized in positive mode. Ion monitoring mode was full scan in the range m/z 50–600. The electron multiplier voltage for detection was 1,350 V.

### Multiple sequence alignment

The coding sequence of GdDODA was compared to betalain-producing enzymes from Caryophyllales plants that have previously been described (S1 Table). DODAs from *G. diazotrophicus* (ID WP_012222467.1), *B. vulgaris* (ID I3PFJ9.1), *Carnegiea gigantea* (ID QED21473.1), *Mesembryanthemum crystallinum* (ID QED21476.1), *Mirabilis jalapa* (ID AJD87536.1), *Phytolacca americana* (ID BAH66635.1) and *Portulaca grandiflora* (ID Q7XA48.1) were employed to perform a multiple sequence alignment by using Clustal Omega (https://www.ebi.ac.uk/jdispatcher/msa/clustalo).

### Statistical analysis

Unpaired t-test for independent means was performed for statistical analysis by using the online Social Science Statistics Calculator (https://www.socscistatistics.com). The significance level for analyzed data was 0.05.

## Results

### Betalain-producing *Nicotiana benthamiana* via expression of betalain biosynthetic pathway genes

To verify whether the intriguing similarity between L-DOPA dioxygenase activities from different kingdoms, such as bacterial and plant origins, make them fully interchangeable, the 4,5-DOPA-extradiol-dioxygenase (DODA) enzyme from *Gluconacetobacter diazotrophicus,* the first bacterium described to produce betalamic acid, was employed to complement the betalain pathway *in planta*. The DODAα1 enzyme from *B. vulgaris* was used as a control and both enzymes were employed to build different plasmids that contain all genes necessary in the betalain pathway (Fig 2). The plasmid pL2SG2 (GdDODA::CYP76AD1::cDOPA5GT) was used to produce betacyanins and pL2HS02 (BvDODAα1::CYP76AD1::cDOPA5GT) was used as its positive control as they express all genes necessary for the biosynthesis of the violet pigments but differ in the nature of DODA enzyme: pL2SG2 expressed DODA from *G. diazotrophicus* and pL2HS02 contained DODAα1 from *Beta vulgaris*. Betaxanthins were obtained through the plasmid pL2SG4 (GdDODA::CYP76AD6), containing the bacterial GdDODA, and pL2SG5 (BvDODA α1::CYP76AD6) harbouring DODAα1 from *B. vulgaris*. In addition, a vector (pL2HS01) expressing only the luciferase gene was employed as a negative control.

### Detection of fluorescent pigments by microscopy

**Fig 3:**
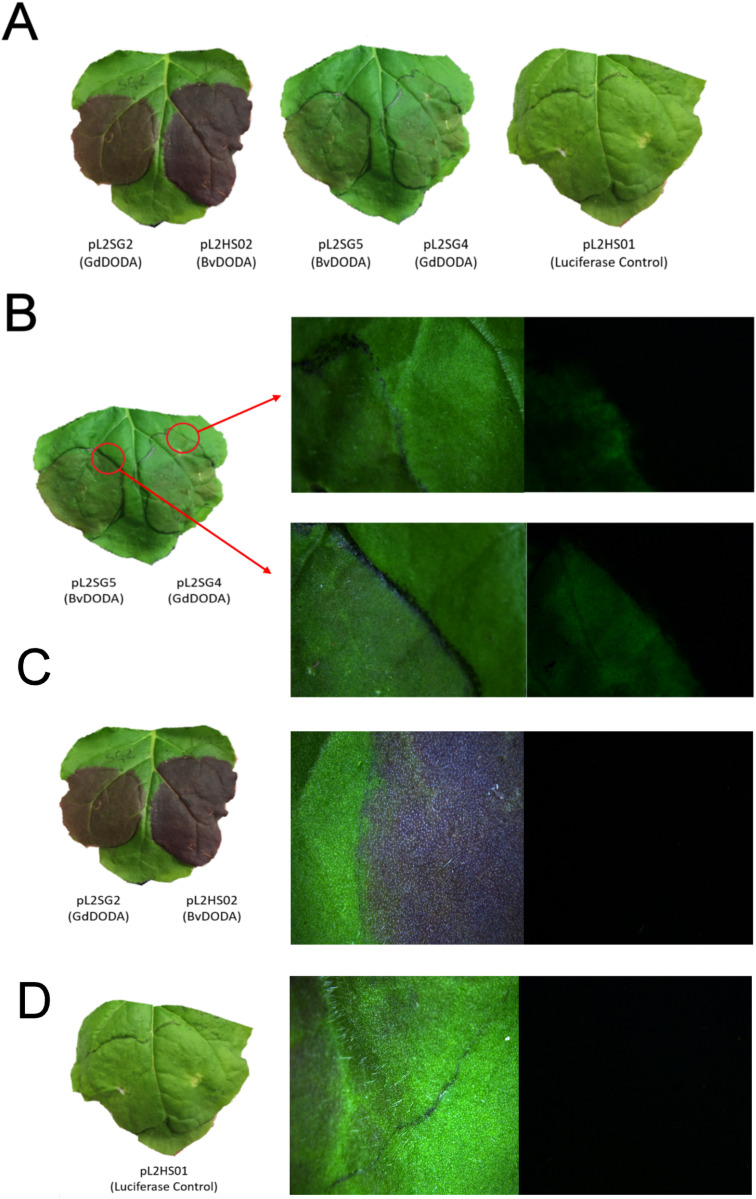
*Nicotiana benthamiana* leaves expressing multigene vectors produce betalains. Images were taken 4 days after infiltration of *Agrobacterium tumefaciens* harbouring the multigene vectors described in Fig 2 (**A**). **B.** Microscopy images showing a closer view of the production of pigments (left) and green fluorescence (right) of leaves infiltrated with pL2SG4 or pL2SG5 due to the presence of betaxanthins. **C.** Lack of fluorescence after agroinfiltration of betacyanin-producing multigene vector pL2HS02. **D.** Lack of fluorescence after agroinfiltration of the control vector pL2HS01.

After infiltration with the betacyanin- and betaxanthin-producing plasmids, we observed the production of pigments in the infiltrated spots after four days. The spots infiltrated with the betacyanin-producing plasmids, pL2SG2 and pL2HS02, produced dark purple spots, while those infiltrated with the betaxanthin-producing plasmids, pL2SG4 and pL2SG5, appeared to have a faint orange colour. The negative control pL2HS01 did not appear to produce any pigments. To confirm the presence of the betaxanthins in *N. benthamiana* leaves, we used the fluorescent properties of these yellow pigments [30]. Samples infiltrated with the different constructs were observed under fluorescence microscopy and images around the infiltration boundary were taken (Fig 3A). Only leaves infiltrated with pL2SG4 or pL2SG5 (Fig 3B) showed fluorescence in the region delimited by the infiltration, indicating the presence of fluorescent compounds obtained after infiltration, presumably betaxanthins. Leaves infiltrated with pLSG2, pL2HS02 or pL2HS01 did not show fluorescence (Fig 3C-D). These initial comparable results between the betacyanin-producing vectors, pL2SG2 and pL2HS02, and between the betaxanthin-producing plasmids, pL2SG4 and pL2SG5, showed that the activity of GdDODA, a bacterial enzyme, could replicate the activity of the plant enzyme, BvDODAα1.

### Spectrophotometric analysis of infiltrated spots

**Fig 4:**
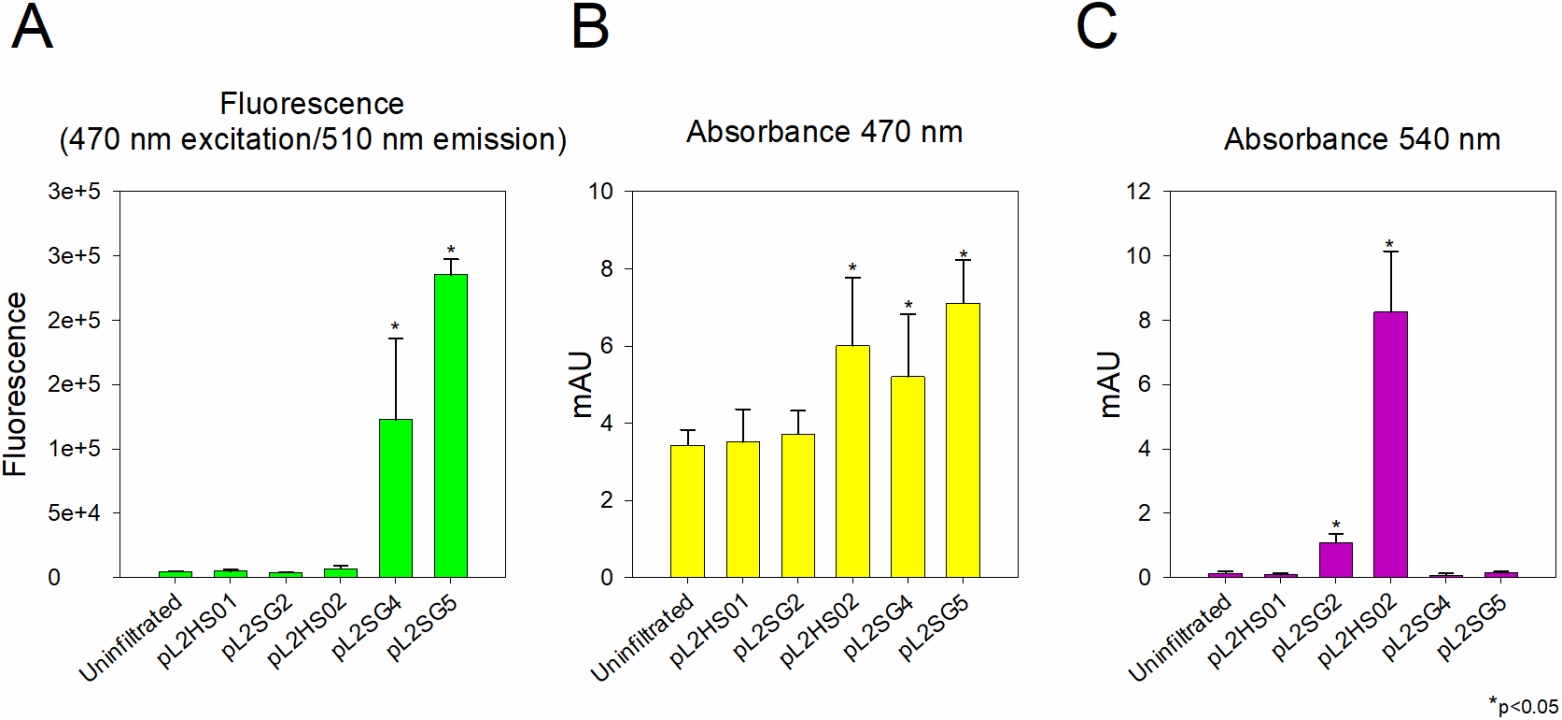
Spectrophotometric properties of extracts from *N. benthamiana* leaves. Mean values of the 5 biological replicates per construct employed in the agroinfiltration assay. **A.** Fluorescence signal of extracts from leaves 4 days after infiltration. The same extracts were employed to measure their absorbance properties at (**B**) λ = 470 nm and (**C**) λ = 540 nm for the detection of betaxanthins and betacyanins, respectively. Asterisks (*) indicate significant differences (p ≤ 0.05) between each experimental sample and the negative control (pL2HS01) as determined by an unpaired t-test.

To further investigate the presence of betacyanins and betaxanthins in infiltration spots, we carried out absorbance and fluorescence measurements. Samples infiltrated with pL2SG4 and pL2SG5 showed fluorescence properties similar to those described for betaxanthins [31], with a maximum excitation wavelength at λ = 470 nm and maximum emission at λ = 510 nm (Fig 4A). Furthermore, absorbance spectra of samples harbouring pL2SG4 (Fig 4B) or pL2SG5 showed a maximum absorbance at λ = 470 nm, similar to that described for betaxanthins. pL2HS02 also showed absorbance at 470 nm but its lack of fluorescence (Figs 3C and 4A) showed that it is not related to the production of betaxanthins. The samples infiltrated with pL2SG2 (Fig 4C) and pL2HS02 were consistent with the biosynthesis of betacyanins because they showed maximum absorbance at λ = 540 nm. Once the presence of betalains in these samples was confirmed, one leaf per construct was selected for further analysis. Quantification of betalains in those leaves was performed using the molar extinction coefficients of ε = 48,000 M-1 cm-1 for betaxanthins and ε  = 65,000 M-1 cm-1 for betanin. Betaxanthin concentration reached a value of 4.11 μmol/mg due to the expression of the bacterial dioxygenase while the expression of BvDODAα1 yielded 29.61 μmol/mg. Regarding betacyanins production, pL2SG2, harbouring GdDODA, produced 1.38 μmol/mg and pL2HS02, which harboured BvDODA, yielded 15.32 μmol/mg (S1 Fig).

### HPLC analysis confirms the presence of betalains in DODA-expressing leaves

In order to further confirm the presence of betalains and explore their diversity in the infiltrated leaves, HPLC analysis was also performed.

**Fig 5:**
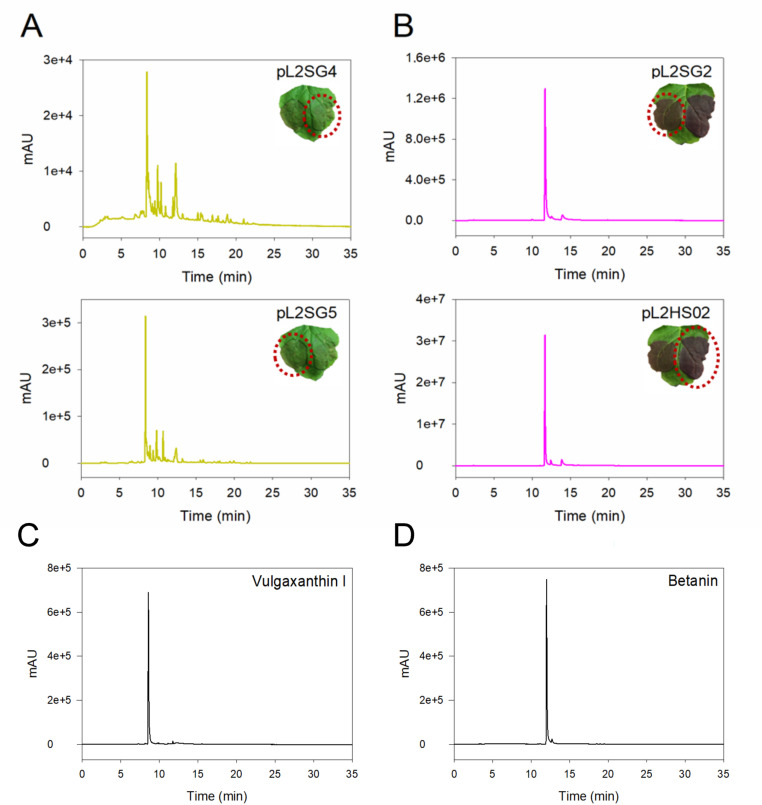
HPLC chromatograms. **A.** Chromatograms at λ = 480 nm from leaves infiltrated with pL2SG4 and pL2SG5. **B.** Chromatograms at λ = 536 nm from leaves infiltrated with pL2SG2 and pL2HS02. **C.** Chromatogram at λ = 480 nm of pure vulgaxanthin I which retention time matches with the main pigment detected by the expression of pL2SG4 and pL2SG5. **D.** Chromatogram at λ = 536 of pure betanin. Retention time matches with the main pigment detected by the expression of pL2SG2 and pL2HS02. Red dotted circles show the part of the leaves analysed.

First, we analysed the analytes present in the samples infiltrated with pL2SG4 and pL2SG5. Samples from the infiltration of pL2SG4 showed two peaks at λ = 405 nm with retention times of 13.38 and 14.92 minutes (S2 Fig). No peak was detected at λ = 536 nm, thus indicating the absence of betacyanins. However, several peaks with different retention times were obtained at λ = 480 nm (Fig 5A), compatible with the presence of betaxanthins. Similar results were obtained in samples from the infiltration of pL2SG5, except for the peak obtained at λ = 405 nm with a retention time of 14.92 minutes. The peak was absent in the leaves that expressed the dioxygenase from *B. vulgaris* but present in the plants transformed with the enzyme from the bacteria *G. diazotrophicus*, this indicates that the peak corresponds to muscaflavin, which has an absorbance maximum at 405 nm, and is produced by the additional 2,3-DOPA-extradiol-dioxygenase activity of GdDODA (S2 Fig). Thus, the expression of pL2SG5 *in planta* is able to produce betaxanthins through the synthesis of betalamic acid as well as pL2SG4, which also produces the additional product muscaflavin.

Then we analysed the analytes from the samples infiltrated with the vectors pL2SG2 and pL2HS02. Samples from the infiltration of pL2SG2 showed the same two peaks that pL2SG4 at λ = 405 nm (betalamic acid and muscaflavin). No peak was detected at λ = 480 nm, indicating the absence of betaxanthins. Three peaks were obtained at λ = 536 nm with retention times of 11.89, 12.52, and 14.31 minutes (Fig 5B), likely corresponding to different types of betacyanins. Analogous results were detected in samples from the infiltration of pL2HS02, except for the peak corresponding to muscaflavin which was absent in these samples.

### Identification of pigments by HPLC-ESI-TOF-MS

HPLC–ESI–TOF-MS analysis was used to unambiguously determine the identity of the compounds obtained from the expression of each plasmid in *N. benthamiana*. Searching for the exact masses of betalamic acid, muscaflavin, betanin, betanidin, and all betaxanthins previously identified in plants [32] was carried out in all samples (S2 Table).

The presence of betalamic acid and muscaflavin was confirmed by the detection of the exact mass of 212.0553 *m*/*z* for the compounds detected at 13.4 and 14.9 minutes. Betalamic acid and muscaflavin were thus confirmed in samples infiltrated with pL2SG2 or pL2SG4, the two plasmids that express the bacterial dioxygenase. Betanin-containing samples infiltrated with pL2SG5 or pL2HS02 also showed the peak corresponding to betalamic acid at 13.4 min.

The exact mass of 552.1586 *m*/*z* was obtained at retention times of 11.89 and 12.52 min in samples infiltrated with pL2SG2 and pL2HS02. These peaks correspond to betanin and its isomer isobetanin, respectively. Their identity was confirmed using pure betanin standards obtained from beetroot extracts. The exact mass of 390.1058 *m*/*z* detected at a retention time of 14.31 min, corresponds to betanidin, and was also obtained in leaves infiltrated with these plasmids. The presence of betanidin was confirmed with pure betanidin obtained from *Lampranthus productus* flowers.

**Fig 6:**
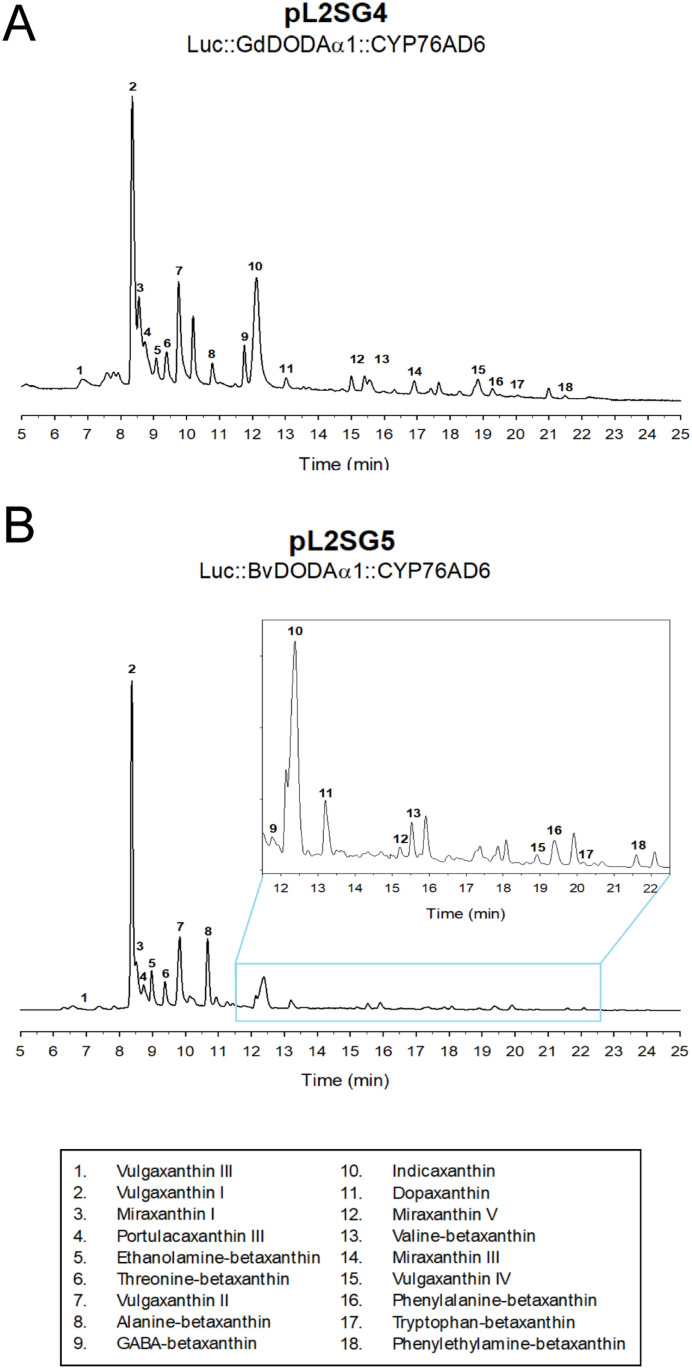
Identification of betaxanthins in leaves infiltrated with pL2SG4 (A) and pL2SG5 (B). The combination of HPLC and HPLC-ESI-MS analysis made possible the identification of the eighteen betalains listed in the box at the bottom of the figure.

Exact masses corresponding to the expected formulae of up to eighteen different betaxanthins were detected in samples infiltrated with pL2SG4 and pL2SG5. Unambiguous determination of the nature of the yellow pigments was attempted by the use of real standards. The biotechnological production of all standards for the possible betaxanthins detected was done individually by biotransformation in *E. coli* (pET28a-GdDODA) cultures [18]. The results obtained with standards coincided in their HPLC retention times with the molecules extracted from the infiltrated leaves. Thus, HPLC analysis and exact masses unequivocally allowed to identify the presence of eighteen betaxanthins, as shown in Fig 6. The major betaxanthin present in all samples was vulgaxanthin I, the betaxanthin derived from the spontaneous condensation of betalamic acid with glutamine [33]. This molecule is one of the main betaxanthins in *B. vulgaris* roots and was obtained here by the engineering of the biosynthetic pathway of the pigments in tobacco, both by using a dioxygenase from the same plant and by using the dioxygenase from the bacterium *G. diazotrophicus*. None of the peaks corresponding to the pigments were detected in samples from the infiltration of the empty vector, showing that the presence of betalains in *N. benthamiana* leaves is strictly related to the recombinant expression of L-DOPA extradiol-dioxygenase enzymes in combination with the other required enzymes of the biosynthetic pathway.

## Discussion

Introducing genes involved in the biosynthetic pathway of betalains in *N. benthamiana* has yielded the heterologous production of a great range of pigments, regardless of the origin of the transgenes [34]. In this work we show that a bacterial DODA enzyme is able to substitute for plant DODA enzymes and produce betalamic acid, and subsequently betalain pigments, *in planta*. It is remarkable that the GdDODA enzyme is adapted to the cellular environment of a Gram-negative prokaryotic cell but can obviously retain its enzymatic activity in the plant cell, despite the different cellular environment and cellular organization. However, our results show the efficiency of the bacterial enzyme does not reach the values obtained when the plant enzyme from *B. vulgaris* was used (Fig 5). While transcription levels were not directly assessed, codon optimization and expression under the same conditions strongly support the assumption of similar protein production rates of GdDODA and BvDODA. Therefore, the lower efficiency of the bacterial enzyme in producing betalains is likely due to its intrinsic properties, with only a minor contribution from production deviations. In our infiltrated leaves, the quantification of betalain content, either betaxanthins or betacyanins, in the route reproduced with the bacterial gene showed a yield one order of magnitude lower than that obtained when BvDODAa1 was employed (Fig 5). This difference in efficiency may be attributed to the difference in cellular conditions, and it may also be related to the presence in GdDODA of dual 2,3- and 4,5-DOPA-dioxygenase activity producing a diversion in the engineered route that yields muscaflavin due to the cleavage of L-DOPA between carbons in position 2 and 3 of the aromatic ring. Previous assays with this enzyme, both *in vitro* and *in vivo*, showed that this is a side activity that represents 10% of its enzymatic activity [17]. Regarding the production of betacyanins, it is noteworthy that the expression of GdDODA or BvDODA resulted in a concentration of betacyanins lower than betaxanthins (S1 Fig). Considering that DODA and CYP76AD1 use L-DOPA as substrate (Fig 1), a lower concentration of betacyanins was expected due to the conversion of L-DOPA to produce cyclo-DOPA by the reaction mediated by CYP76AD1, decreasing the concentration of L-DOPA that DODA can use as substrate to produce betalamic acid.

While GdDODA is less efficient in the production of betalains than BvDODAa1 under our conditions, it is still efficient in complementing a plant biosynthetic route since the profile of pigments was equivalent between GdDODA and BvDODAα1-transformed tissue (S2 Table), apart from the addition of muscaflavin in the GdDODA assays. The expression of DODA enzymes in *N. benthamiana* leaves has typically been compared by measuring betanin content since this betalain produces a violet coloration easily detectable by the naked eye in contrast to the detection of yellow betaxanthins, whose production is hidden by the green color of the leaves. We identified the betacyanins produced from the constructs combining CYP76AD1 and the respective DODAs, but we also identified the betaxanthins by fluorescence microscopy, HPLC and HPLC–ESI–TOF-MS, representing the first time that the betaxanthins have been individually identified in betalain-producing *N. benthamiana* leaves that expressed the constructs that combined CYP76AD6 and DODA. Although pL2HS02-infiltrated leaves initially showed absorbance at 470 nm, fluorescence microscopy and HPLC-ESI-TOF-MS confirmed the absence of betaxanthins and the production of the betacyanins betanin, isobetanin and betanidin (S2 Table). These analyses showed that both enzymes, BvDODA and GdDODA, produced the same set of betalains with vulgaxanthin I, the betaxanthin derived from L-glutamine, being the main pigment obtained (S2 Table). The betalain profile depends on the amino acid content of the species [35] so the great amount of vulgaxanthin I obtained with respect to the rest of betaxanthins indicates a high content of L-glutamine in *N. benthamiana*. The presence/absence of 2,3-seco-DOPA caused by the additional 2,3-DOPA-dioxygenase activity of GdDODA does not alter the pigment profile because subsequent cyclization of the intermediate and condensation with free amino groups are spontaneous reactions. Overall, obtaining the same pattern of betalains indicates that the two DODA enzymes participated in the cleavage of L-DOPA to give rise to the intermediate 4,5-seco-DOPA independently of their origin, sequence homology and side activities.

DODA enzymes with high betalamic acid-forming activity described in plants have been suggested to arise after duplication and neofunctionalization events [13] from ancestral DODA enzymes with unknown activity that may or may not be related to betalain formation. These enzymes have been identified across all plants and are known as LigB enzymes due to their structural similarity with the LigB domain of bacterial extradiol 4,5-dioxygenases [36,37]. GdDODA is predicted to show some local structural homology to the solved structure of *Escherichia coli* ygiD, a LigB enzyme [17,38] suggesting that a similar reaction mechanism may be involved. Between YgiD and the plant DODAs, PgDODA, MjDODA and BvDODA, three strictly conserved histidine residues have been identified that are likely involved in metal coordination in YgiD (His22, His57, and His234) and these are considered essential for L-DOPA cleavage activity [37]. The His234 was previously identified in GdDODA as His101 [17]. It is flanked upstream by an amino acid with acid charge and downstream by one with hydrophobic charge, as it is described for plant DODA enzymes [38]. Additionally, His173 inside of the conserved motif H-P-S/AN/D-x-T-P in betalain-forming DODAs from plant species has been suggested to be essential for their catalytic activity [37]. Sequence alignment from this conserved block does not show significant sequence similarity between GdDODA and plant DODA enzymes (S3 Fig). Given the small size of GdDODA compared to BvDODA, it is also remarkable how a bacterial enzyme with only 15.60 KDa is able to recreate the activity of plant enzymes that have an average size of 31 KDa. Furthermore, GdDODA is a dimer protein under native conditions [17] whereas little is known about the native state of plant DODAα1 enzymes. The paralogue protein DODAα2 from *B. vulgaris* [19] and *Chenopodium quinoa* [39] have shown to be monomers under native conditions but the lack of information about DODAα1, which is indeed responsible for the production of betalains in plants, make it difficult to correlate the protein dimerization to the ability to produce betalains. In a similar manner, the recently obtained crystal structure of BvDODAα2 [40] does not allow for establishing an activity-structure relationship since the ability of this paralog protein to produce betalains is scarce [41]. Altogether, our results show that further analysis is necessary in terms of molecular mechanisms and three-dimensional disposition of amino acids to determine whether any convergence in activity and reaction mechanism between plant DODAs and GdDODA is due to the conservation of structural elements from a common ancestor protein, or due to the evolution of convergent structural features from different proteins. Convergence of enzymatic activity despite little sequence similarity is entirely possible for these enzymes as there are many examples in nature that show the existence of different enzymes with no significant similarity but sharing the same catalytic reaction [42] probably due to a structural similarities. For instance, human and archaeal carbonate dehydratases have converged to produce hydrogencarbonate through the nucleophilic attack of a hydroxide ion on carbon dioxide [43]. These two enzymes employ different metal ions as cofactor but share three histidine residues in the active site that coordinate the catalytic reaction. Mammalian and viral lysozymes only show local sequence alignment of three acidic residues (DDE) at their active site but yet catalyze the hydrolysis of glycosidic bonds in peptidoglycan [44]. More interesting is the case of licheninases that produce cell wall degradation. This enzyme is found in plants and bacteria with unrelated primary sequence and tertiary structure. Despite this lack of sequence similarity, they cleave β-D-glucans and lichenin by producing a covalent bond between the substrate and a glutamic acid residue in the active site of the enzymes [45]. Given this functional convergence of enzymes from different kingdoms, convergency of plant and bacterial DODA is a plausible explanation of the shared ability to produce betalains.

## Conclusions

The analyses employed here show that transient expression of betalains in *N. benthamiana* is possible not only through the expression of plant enzymes but also by employing an enzyme from a prokaryotic organism with a completely different origin, structure and with an additional enzymatic activity. Differences in the sequences of the enzymes here employed support that convergent evolution underlies the appearance of betalain-producing enzymes and the lack of similarities shows the great plasticity that betalain-forming dioxygenase enzymes present.

## Supporting information

S1 Fig Betalain content of *N. benthamiana* leaves expressing betalain-producing genes. (PDF)

S2 Fig HPLC chromatograms at λ = 405 nm from leaves infiltrated in this work. Green rectangle contains betalamic acid and blue rectangle contains muscaflavin.
(TIF)

S3 Fig Multiple sequence alignment of DODA enzymes. Sequence comparison of the catalytic region that involves the highly conserved histidine present in betalain-producing enzymes (red), including GdDODA.
(PDF)

S1 Table Accession number of genes employed in this work. (PDF)

S2 Table List of compounds screened in leaves infiltrated with the different constructs employed in this work. Detected compounds are marked with a X in the corresponding samples.
(PDF)
