# Supplementary figures and images for: In planta complementation of the betalain biosynthetic pathway with a bacterial dioxygenase
Source: PLoS One. 2025 Jun 24;20(6):e0325603. doi: 10.1371/journal.pone.0325603 (PMC12186922; doi:10.1371/journal.pone.0325603)

Figure S1. Betalain content of *N. benthamiana* leaves expressing betalain-producing genes.

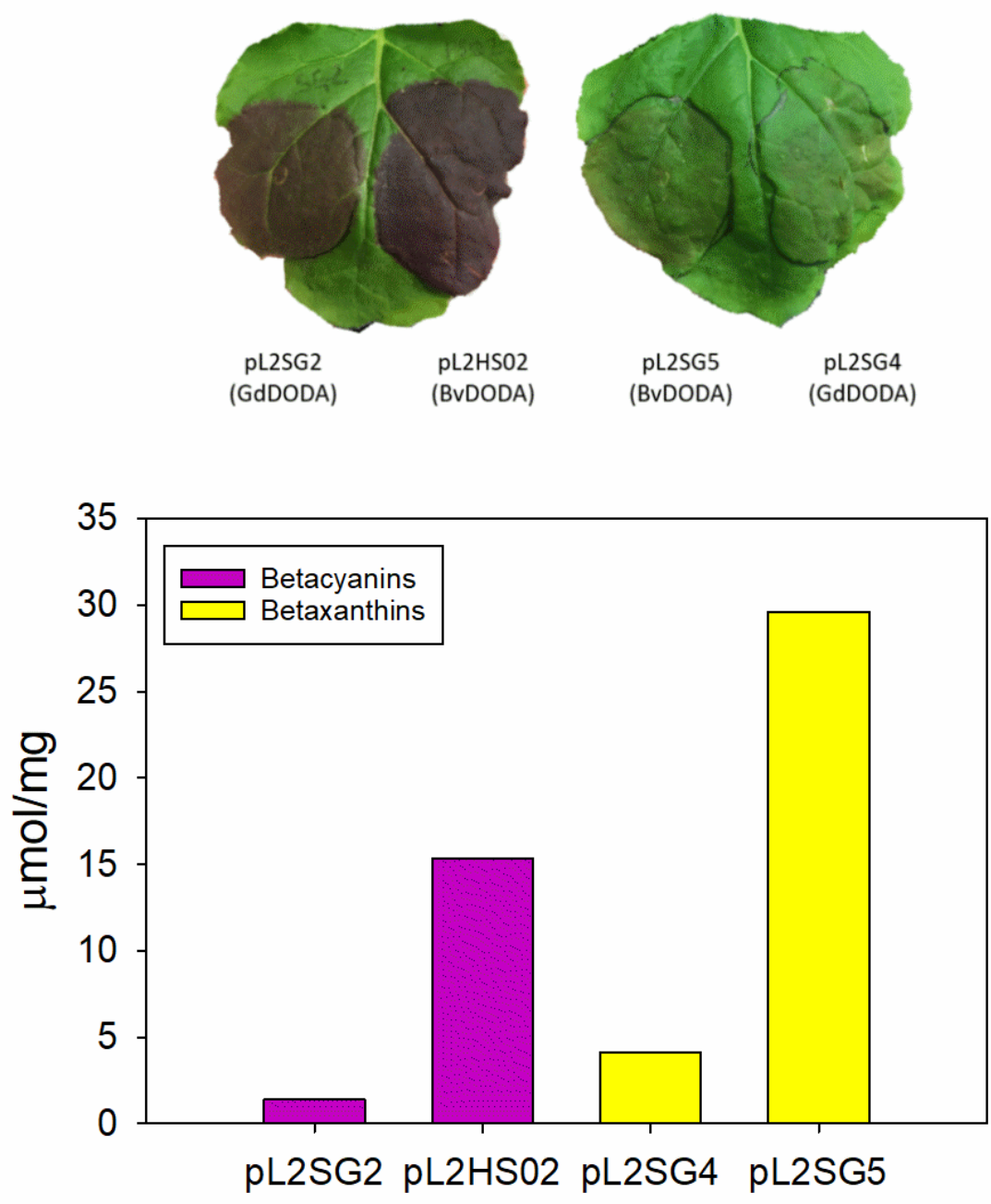

Supplement: S1 Fig — (PDF) [file pone.0325603.s001.pdf]
