# Supplementary material for: In planta complementation of the betalain biosynthetic pathway with a bacterial dioxygenase
Source: PLoS One. 2025 Jun 24;20(6):e0325603. doi: 10.1371/journal.pone.0325603 (PMC12186922; doi:10.1371/journal.pone.0325603)
Supplement: S3 Fig — Sequence comparison of the catalytic region that involves the highly conserved histidine present in betalain-producing enzymes (red), including GdDODA. (PDF) [file pone.0325603.s003.pdf]

```
GdDODA    -------HLRD---------GIWLGQPRALLGSRLPEDAAEAD----GAGTPDTTPGGATPI-----------------------------------------
BvDODA    HPLDETPHYFDGVAPWAAAFDSWLRK--ALINGRFEEVNIYESKAP-NWKLAHPFPEHFYPLHVVLGAAGEKWKAELIHSSWD-HGTLCHGSYKFTSA-----
CgDODA    HPSDDTPHWFDGVAPWAAEFDQWLED--ALINGRYDDVNNYQTKAPSGWKIAHPIPEHFLPLHVAMGAAGEKSKAELIYRTWD-HGTLGYASYKFTSI-----
McDODA    HPSDDTPHCPNAVAPWAAEFDDWLCD--AVIKGRYEDVNNYNKLAP-NWEIAHPGPEHLYPLHVALGAAGEKSIAETIHHSWARNGVFGYASFKFTSTSSTL-
MjDODA    HPSDDTPHCPNGVAPWAIEFDNWLED--ALLSGRYEDVNNFKKLAP-NWEISHPGQEHLYPLHVALGAAGKNPKTQLIHRSWAANGVFGYSTYNFTPTTQKTD
PaDODA    HPSNDTPHA-VGVAPWAAEFDNWLEE--ALTSGRYEDVNNYQTKAP-NWKIAHPWPEHFYPLHVAMGAAGENAKAELIHRSWE-HGTLGYACYKFTSS-----
PgDODA    HPSDDTPHWFDGVAPWAAEFDQWLED--ALLEGRYEDVNNYQTKAPEGWKLAHPIPEHFLPLHVAMGAGGEKSKAELIYRTWD-HGTLGYASYKFTSI-----
```
