# Supplementary material for: In planta complementation of the betalain biosynthetic pathway with a bacterial dioxygenase
Source: PLoS One. 2025 Jun 24;20(6):e0325603. doi: 10.1371/journal.pone.0325603 (PMC12186922; doi:10.1371/journal.pone.0325603)
Supplement: S1 Table — (PDF) [file pone.0325603.s004.pdf]

S1 Table. Accession number of genes employed in this work.

**Gene name**	**Sequence ID**
Gluconacetobacter diazotrophicus DODA	WP_012222467.1
Beta vulgaris DODA	I3PFJ9.1
Carnegiea gigantea DODA	QED21473.1
Mesembryanthemum crystallinum DODA	QED21476.1
Mirabilis jalapa DODA	AJD87536.1
Phytolacca americana DODA	BAH66635.1
Portulaca grandiflora	Q7XA48.1
Photinus pyralis LUC	M15077
Beta vulgaris CYP76AD1	MH836617
Beta vulgaris CYP76AD6	KT962274
Mirabilis jalapa cDOPA5GT	MH836618
