# Supplementary material for: In planta complementation of the betalain biosynthetic pathway with a bacterial dioxygenase
Source: PLoS One. 2025 Jun 24;20(6):e0325603. doi: 10.1371/journal.pone.0325603 (PMC12186922; doi:10.1371/journal.pone.0325603)
Supplement: S2 Table — Detected compounds are marked with a X in the corresponding samples. (PDF) [file pone.0325603.s005.pdf]

S2 Table. List of compounds screened in leaves infiltrated with the different constructs employed in this work. Detected compounds are marked with a X in the corresponding sample.

				pL2SG4	pL2SG5	pL2SG2	pL2HS02	pL2HS01	Uninfiltrated
**Compound**	**Chemical formula**	**Calculated mass (m/z+1) (theoretical)**	**TOF Exact mass (m/z+1) (experimental)**						
Betalamic acid	$C_9H_9NO_5$	212,0481	212,0553	x	x	x	x		
Muscaflavin	$C_9H_9NO_5$	212,0481	212,0553	x		x			
Ethanolamine betaxanthin	$C_{11}H_{14}N_2O_5$	255,0903	255,0975	x	x				
Portulacaxanthin III	$C_{11}H_{12}N_2O_6$	269,0695	269,0768	x	x				
Putrescine betaxanthin	$C_{13}H_{19}N_3O_4$	282,1375	282,1448						
Alanine betaxanthin	$C_{12}H_{14}N_2O_6$	283,0852	283,0925	x	x				
Serine betaxanthin	$C_{11}H_{12}N_2O_7$	285,0644	285,0717						
GABA-betaxanthin	$C_{13}H_{16}N_2O_6$	297,1008	297,1081	x	x				
Histamine betaxanthin	$C_{14}H_{16}N_4O_4$	305,1172	305,1244						
Indicaxanthin	$C_{14}H_{17}N_2O_6$	310,1087	310,1159	x	x				
Valine betaxanthin	$C_{14}H_{18}N_2O_6$	311,1165	311,1238	x	x				
Threonine betaxanthin	$C_{13}H_{16}N_2O_7$	313,0958	313,103	x	x				
Phenylethylamine betaxanthin	$C_{17}H_{18}N_2O_4$	315,1266	315,1339	x	x				
Vulgaxanthin IV	$C_{15}H_{20}N_2O_6$	325,1321	325,1394	x	x				
Vulgaxanthin III	$C_{13}H_{15}N_3O_7$	326,0909	326,0983	x	x				
Portulacaxanthin I	$C_{14}H_{17}N_2O_7$	326,1035	326,1109						

Humilixanthin	$C_{14}H_{18}N_2O_7$	327,1114	327,0823						
Miraxanthin II	$C_{13}H_{14}N_2O_8$	327,075	327,1187						
Miraxanthin III	$C_{17}H_{18}N_2O_5$	331,1216	331,1288	x	x				
Vulgaxanthin I	$C_{14}H_{17}N_3O_7$	340,1066	340,1139	x	x				
Lysine betaxanthin	$C_{15}H_{21}N_3O_6$	340,143	340,1503						
Vulgaxanthin II	$C_{14}H_{16}N_2O_8$	341,0906	341,0979	x	x				
Methionine betaxanthin	$C_{14}H_{18}N_2O_6S$	343,0886	343,0958						
Miraxanthin V	$C_{17}H_{18}N_2O_6$	347,1165	347,1238	x	x				
Muscaaurin VII	$C_{15}H_{16}N_4O_6$	349,107	349,1143						
Miraxanthin I	$C_{14}H_{18}N_2O_7S$	359,0835	359,0907	x	x				
Phenylalanine betaxanthin	$C_{18}H_{18}N_2O_6$	359,1165	359,1238	x	x				
3-Methoxytyramine betaxanthin	$C_{18}H_{20}N_2O_6$	361,1321	361,1394						
Arginine betaxanthin	$C_{15}H_{21}N_5O_6$	368,1492	368,1565						
Portulacaxanthin II	$C_{18}H_{18}N_2O_7$	375,1114	375,1187						
Betanidin	$C_{18}H_{17}N_2O_8$	390,0985	390,1058			x	x		
Dopaxanthin	$C_{18}H_{18}N_2O_8$	391,1063	391,1136	x	x				
Tryptophan betaxanthin	$C_{20}H_{20}N_3O_6$	399,1352	399,1425	x	x				
Betanin	$C_{24}H_{27}N_2O_{13}$	552,1513	552,1586			x	x		
Isobetanin	$C_{24}H_{27}N_2O_{13}$	552,1513	552,1586			x	x		
